# Supplementary material for: Pre-Dialysis B-Line Quantification at Lung Ultrasound Is a Useful Method for Evaluating the Dry Weight and Predicting the Risk of Intradialytic Hypotension
Source: Diagnostics (Basel). 2022 Nov 29;12(12):2990. doi: 10.3390/diagnostics12122990 (PMC9776885; doi:10.3390/diagnostics12122990)

Supplementary Figure S1. ROC curves according to the NYHA class.

A different number of pre-dialysis B-lines predicts IDH in patients with mild and severe heart failure. A subgroup analysis was conducted according to the NYHA class, comparing patients with mild heart failure (NYHA class 0–2) and severe heart failure (NYHA class 3–4). Binary logistic regression confirmed the role of pre-dialysis B-line scores in predicting IDH episodes (NYHA class 0–2: p = 0,012; NYHA class 3–4: p = 0,04). ROC analysis was then performed, and the area under the ROC curve was 0,747 (CI 95% 0,634 - 0,859) for patients with NYHA class 0–2 (Supplementary Figure S**1A**) and 0,873 (CI 95% 0,724 - 1,0) for patients with NYHA class 3–4 (Supplementary Figure S**1B**). According to the Youden index method, in patients with NYHA class 0–2, the optimal threshold to predict an IDH episode was reached when 8 B-lines were detected (sensitivity 70,5%, specificity 75%), while in patients with NYHA class 3–4, the optimal threshold was reached when 20 B-lines were detected (sensitivity 92,3%, specificity 84,6%).

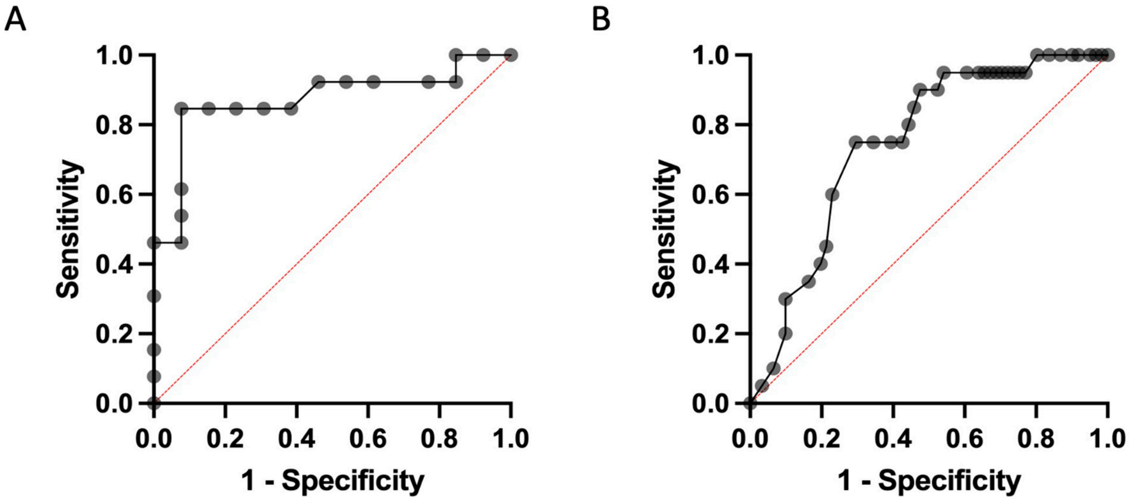

Supplement: Supplementary file 1 [file diagnostics-12-02990-s001.zip › diagnostics-1970483-supplementary.pdf]
